# Adolescent gender norms and adult health outcomes in the USA: a prospective cohort study

**DOI:** 10.1016/S2352-4642(19)30160-9

**Published:** 2019-08

**Authors:** Holly B Shakya, Ben Domingue, Jason M Nagata, Beniamino Cislaghi, Ann Weber, Gary L Darmstadt

**Affiliations:** a Center on Gender Equity and Health, Division of Infectious Disease and Global Public Health, University of California San Diego, San Diego, CA, USA; b Department of Education, Stanford University, Stanford, CA, USA; c Department of Pediatrics, School of Medicine, Stanford University, Stanford, CA, USA; d Department of Global Health and Development, London School of Hygiene and Tropical Medicine, London, UK

## Abstract

**Background:**

Previous research has documented differences in health behaviours between men and women, with differential risks and health outcomes between the sexes. Although some sex-specific differences in health outcomes are caused by biological factors, many others are socially driven through gender norms. We therefore aimed to assess whether gender expression as an adolescent, determined by the degree to which an individual's behvaiours were typical of their gender, were associated with health behaviours and outcomes in adulthood.

**Methods:**

In this prospective cohort study, we used data from the National Longitudinal Study of Adolescent to Adult Health, a nationally representative sample of US adolescents from whom data were collected during adolescence (ages 11–18 years) and adulthood (ages 24–32 years). We created a measure of gender expression that was based on the degree to which male and female adolescents and adults behave in stereotypically masculine (for men) or feminine (for women) ways relative to their same-gender peers. Adolescents were assessed for baseline sociodemographic characteristics and gender expression, and these participants were later assessed, during adulthood, for their gender expression and health behaviours and outcomes, which included depression, self-rated health, drug and alcohol use, cardiovascular risk factors, experience of sexual violence, diet, and obesity. These data were collected via surveys, except for body-mass index, cholesterol, and blood pressure, which were collected as biomarkers.

**Findings:**

Between April and December, 1995, self-reported data were collected from 10 480 female and 10 263 male adolescents; similar data were subsequently collected in several waves in this cohort, with a final collection between January, 2008, and February, 2009, when participants were aged 24–32 years. We used data from this final wave and from baseline, and our study represents a secondary analysis of these data. Of these participants, complete follow-up data from 6721 (80%) adult women and 5885 (80%) adult men were available. Gender expression was stable for men and women from adolescence to adulthood. High masculinity (*vs* low masculinity) in adolescent and adult men was positively associated with smoking in the past month, use of marijuana and recreational drugs, prescription drug misuse (adult gender expression only), and consumption of fast food and soda (adolescent gender expression only) in the past week. However, higher masculine gender expression in adult men was negatively associated with diagnosed depression and high cholesterol in adulthood, and masculine gender expression in adolescent and adult men was negatively associated with high blood pressure in adults. High femininity (*vs* low femininity) in adolescent or adult women was positively associated with high cholesterol and blood pressure (both adult gender expression only), depression, migraines (adult gender expression only), and physical limitations (ie, health problems that limited their daily activities). However, higher femininity in adolescence was negatively associated with self-rated good health in adulthood. Although feminine gender expression in adolescents was predictive of adult recreational and prescription drug and marijuana use and experience of sexual violence, feminine gender expression in adulthood was negatively associated with adult substance use and experience of sexual violence, suggesting that expressions of femininity typical of adolescents impart risks that expression of femininity as an adult does not. Individuals who are highly masculine or feminine seem to be at greatest risk of adverse health outcomes and behaviours.

**Interpretation:**

We found compelling evidence that adolescent gender expression is correlated with health in adulthood independently of gender expression as an adult. Although more research is needed to identify causal mechanisms, our results suggest that those designing health behaviour interventions should carefully consider integrating gender transformative components into interventions.

**Funding:**

Eunice Kennedy Shriver National Institute of Child Health and Human Development, Gender Equality, Integrated Delivery, HIV, Nutrition, Family Planning, and Water Sanitation and Hygiene Program Strategy Teams (Bill and Melinda Gates Foundation).

## Introduction

A large body of research has documented differences in health behaviours between men and women, and differential risks of several health outcomes. Men (including male adolescents), for instance, are more likely than women to smoke, binge drink, use marijuana and recreational drugs, and abuse prescription drugs.^1^, ^2^, ^3^ Men are also less likely to use preventive care services^4^ and more likely to engage in crime and risky behaviour.^5^, ^6^ Women (including female adolescents), by contrast, have higher rates of depression^7^ and are less likely to engage in physical activity.^8^ Previous studies^9^ on stress and coping identified distinct gender differences in coping mechanisms, finding that men were less emotionally expressive and women were more prone to psychological distress. Although some sex-specific differential health outcomes are caused by biological factors (for instance, men cannot get ovarian cancer and women cannot get prostate cancer), other differences in health outcomes are socially driven by restrictive gender norms or societal expectations of what is appropriate for men and for women.^3^, ^9^, ^10^, ^11^, ^12^ Gender norms are cultural schemas of what is masculine and what is feminine and, although some gender norms cross cultural boundaries, others are contextually specific. Behaviours that are driven by gender norms differ in their expression between those who are biologically male and female, and the cost of not conforming to those norms will also differ by the extent to which the norms are a salient factor in each specific context. An understanding of how gender norms contribute to differential health outcomes can be crucial for efforts, both at the population level and at the clinical level, to reduce morbidity and mortality from preventable causes.^13^, ^14^

**Research in context**

**Evidence before this study**

Differences in health behaviour between men and women and the differential health outcomes that result have been well documented through years of research. On average, men (including male adolescents) are more likely to misuse substances, are less proactive regarding their health, are less likely to use preventive care services, and are more likely to engage in risky behaviour (eg, not using a seatbelt, smoking and drinking to excess, committing crimes). By contrast, women do less physical activity and are more likely to be diagnosed with depression. Many of these differences are driven by socially reinforced gender norms—societal expectations of the ideal man and the ideal woman—but to what extent these norms affect gendered differences is unclear. There is evidence that gender norms among adolescents are taught by parents and reinforced through peers. Adolescents who do not conform to the respective gender norms of their biological sex, either masculine for males or feminine for females, are often ostracised and bullied. The consequences of this type of sanctioning can be associated with a higher risk of depressive symptoms in adolescence that persists into adulthood. Although there is evidence that adherence to highly masculine or highly feminine expectations imparts health risks, it is unclear whether these gender-related risks persist regardless of the individual's biological sex. We did a comprehensive literature search of studies on gender norms and health published before December 31, 2018, with Google Scholar. We also investigated references found in relevant work. We used the search terms “gender norms health”, “gender norms over time”, “masculinity health”, “femininity health”, “gender norms mental health”, “masculinity risks”, and “femininity risks”. Relevant studies came from a range of disciplines including sociology, psychology, social psychology, and public health, and they were published between 1997 and 2018. We found no studies that used a context-specific behaviourally based measure of gender that tracked the association of gender expression over time from adolescence to adult health outcomes in both genders.

**Added value of this study**

We found that a person's gender-specific behaviours as an adolescent were associated with their health and health behaviours into adulthood. Although it is well established that childhood experiences, such as trauma or neglect, can affect future health, to our knowledge, this is the first analysis to show that a person's gender expression toward masculinity or femininity can track through time to affect their health in adulthood, including the likelihood of engaging in risky health behaviours and of several health outcomes. Whether that effect is protective or harmful is dependent on the gender of the individual, their degree of gender expression, and the behaviour in question. We found evidence that, for some outcomes, masculinity and femininity convey risks independent of whether a person is biologically male or female.

**Implications of all the available evidence**

Our results suggest that addressing gender expression could be a relevant strategy for health behaviour interventions and that, for some behaviours, a strong orientation towards masculinity or femininity matters regardless of whether a person is biologically male or female. Future research should investigate causal pathways by which these effects occur.

There is evidence that gender norms among adolescents are at least partly inculcated by parents and reinforced through peers.^15^, ^16^ Adolescents who are non-conformant to their respective gender norms are often ostracised and bullied^15^, ^17^ and such sanctioning can be associated with higher risk of depressive symptoms in adolescence that persist into adulthood.^18^ Although gender norms affect health for all those on the spectrum of gender, in some contexts, challenging gender norms can be more difficult for boys because girls experience more leniency in behaving in more stereotypically masculine ways, whereas boys are heavily penalised for expressions of femininity.^15^, ^18^, ^19^

Courtenay^19^ asserts that, in the USA, specific behavioural expressions of masculinity put men at heightened risk of compromised health because of the perceived necessity of conforming to stereotypical concepts of independence, self-reliance, strength, and stoicism that are associated with masculinity. A systematic review^1^ of literature on women's substance use found mixed results regarding the degree to which women adhered to feminine behavioural norms and the associated risk of substance use. There was some evidence that so-called emotionality put women at risk for heavy episodic drinking although, in other contexts, heavy drinking was considered at odds with femininity, resulting in a negative correlation. Wilkinson and colleagues^20^ found evidence that increases in gender-specific behavioural expressions (ie, increased stereotypical masculine behaviours in men and feminine behaviours in women) over time were positively associated with substance use in men and negatively associated with substance use in women. Another study^21^ demonstrated that men who were higher on a masculinity scale (comprising typically masculine attitudes and behaviours) were less likely to engage in health promoting behaviours and more likely to engage in risk-taking behaviours. These studies suggest that masculinity and femininity might impart health risks; however, it is unclear whether these gender-related risks persist regardless of the individual's biological sex—eg, whether masculinity is risky both for men and for women.

In our study, we adapted a constructed measure of gender expression^22^, ^23^—ie, the extent to which an individual engages in behaviours and attitudes considered typical of males (masculine) or females (feminine), independent of their sexual orientation or the gender to which they feel romantically and sexually attracted. This approach represents a different method of measuring gender compared with scales such as the Bem Sex Role Inventory,^24^ for which the questions remain the same over time. The gender expression measure was developed by Cleveland and colleagues^23^ to examine genetic differences in gender-related behaviours, applied by Nowotny and colleagues^25^ to evaluate the association between gendered behaviours and suicidal ideation, and further refined and applied by Fleming and colleagues^22^ to analysis of violent behaviour and substance use.^20^ The gender expression measure is calculated with behaviours and attitudes considered typical of individuals of a given sex and a given age and time period. With longitudinal measures of gender expression, it is possible to consider the long-term effects of gender expression on health.

It is well known that childhood and adolescent experiences can affect adult health; for instance, exposure to trauma or neglect and childhood and adolescent experiences of low socioeconomic status can negatively affect adult health.^26^, ^27^ Research^2^, ^17^ also suggests that not behaving in a way typical of one's gender as an adolescent can negatively affect adult health, perhaps through similar mechanisms involving allostatic load or stress. We therefore aimed to investigate three fundamental, relatively unexplored questions. First, does an individual's level of gender expression persist from adolescence to adulthood? Although some research^1^, ^19^, ^20^, ^21^ has measured the association between gender expression and health, we know of no studies that examine the permanence of gender expression over time. Second, does adolescent gender expression predict adult health outcomes? Finally, across the spectrum of masculinity and femininity for males and females, how is adult gender expression associated with adult health outcomes?

## Methods

### Study design and participants

In this prospective cohort study, we used data from the National Longitudinal Study of Adolescent to Adult Health cohort, a nationally representative sample of US adolescents who were followed up into adulthood. The baseline sample (wave 1; at ages 11–18 years), who were interviewed in their homes, was recruited from 80 randomly selected high schools and paired middle schools across the USA.^28^ The participants were followed up in serial face-to-face interviews through four waves of data collection, with wave 4 occurring at ages 24–32 years.

At baseline and follow-up, an interviewer travelled to the home of or another suitable location for the potential participant. Interviews lasted approximately 90 mins and were conducted in an area deemed to be as private as possible. Audio computer-assisted self-reports (at baseline) and computer-assisted self-reports (at follow-up) were used by participants to answer potentially sensitive questions.

Written consent was obtained from a parent, if the participant was younger than 18 years, or from the participant if the participant was 18 years or older. The University of North Carolina Institutional Review Board approved all Add Health study procedures.

### Independent variables: degree of gender expression

The primary exposure variable was a measure of a participant's gender expression.^22^, ^23^ The gender expression measure assessed the degree to which men and women behave in ways that are similar to the behaviours of their same-sex peers by use of the variables that best discriminate between men and women; these variables were found by use of *t* tests to identify those variables that show the most significant differences between genders (appendix).^22^ Because this measure is based on behaviour, it was designed to capture the performance of gender rather than self-reported ideologies or attitudes towards gender-specific social expectations. We calculated the measure from the variables identified by Fleming and colleagues^22^ for waves 1 (25 items) and 4 (22 items), but we omitted variables that were measuring the same characteristics as our outcomes. For instance, when we used gender expression to predict depression, we removed any variables from the gender expression measure calculation that were specific to depression.

The behavioural measures were used in a logistic regression model to create a predicted probability of being a male or female. We first used responses from those individuals outside an individual's school to fit the model, and then we projected probabilities for those individuals inside the school with these estimated parameters. Predicted probabilities were estimated for the full set of items, not for each of the individual items. No covariates were used in the prediction of gender; the full model only comprised the responses to the included items.

To capture the potential effects from the school environment, we included a school-level aggregate measure from wave 1 gender expression in our models. All gender expression variables were Z score-standardised for ease of interpretation. A high gender expression score for women represents more feminine behaviours and a high gender expression score for men represents more masculine behaviours, whereas men with lower gender expression scores engage in typically more feminine behaviours, while women with lower gender expression scores engage in typically more masculine behaviours. The behaviours used in the scale were those identified as being typical of boys or girls; neutral behaviours were not included. The associations between gender expression and the outcomes of interest could be non-linear because we cannot assume that the relationship between a health outcome, such as self-rated health, and masculinity, for instance, is the same between high levels of masculinity and average levels as it between average levels and low levels. To capture possible non-linearity, we also created categories of gender expression by use of gender-specific tertiles.

### Dependent variables: health outcomes and health behaviours

Our dependent variables were wave 4 (ie, adulthood) health outcomes and health behaviours (appendix), which were predominantly binary measures. We collected self-reported data on whether each participant had experienced sexual violence; had been diagnosed with high cholesterol, high blood pressure, or depression; had physical limitations (ie, problems that limited their daily activities) caused by a health condition; had ever used marijuana or recreational drugs or misused prescription drugs; had become “very drunk” in the past year; had smoked any cigarettes within the past month; had consumed soda within the past week; or had eaten fast food within the past week. We also collected binary data on whether each participant was obese, which was based on body-mass index measurements and, separately, on whether they had high cholesterol or high blood pressure, which were based on the survey biomarker tests (anthropometric and cardiovascular measurements collected by the interviewers after the survey). Continuous measures included self-rated health, a past-week physical activities index, and depression, which was measured by responses to the Center for Epidemiologic Studies Depression Scale (CESD), regarding the participant's experiences of the previous week.

### Control variables

We included a continuous measure of the participant's age at wave 1, non-exclusive binary measures for ethnicity (including Asian, black, and Hispanic) and a constructed socioeconomic status variable (appendix).^29^ We also used a measure of sexual orientation, which was taken at wave 4.

### Statistical analysis

Linear regressions were first used to test whether gender expression at wave 1 was predictive of gender expression at wave 4. Either logistic or linear regression (depending on the outcome variable) was used to test whether wave 1 gender expression and wave 4 gender expression were predictive of wave 4 health outcomes and health behaviours. As a post-hoc specification, we ran two additional separate sets of models for each outcome by use of categorical versions of gender expression that was split into tertiles, with one model comparing the highest and middle tertiles to the lowest tertile (ie, those who were most or a median level of masculine [men only] or feminine [women only] compared with those who were least masculine [men] or feminine [women]), and another model comparing the higher and lower tertiles to the middle tertile. Wave 1 gender expression and wave 4 gender expression were included together in all models, which were stratified by sex, included demographic variables as controls, and were run with the appropriate survey weighting and clustering on the school level. Participants with missing data were omitted from the analysis. To aid in visualisation and to identify potentially non-linear trends, we created plots for each model using locally weighted smoothing. Our statistical significance cutoff threshold was 0·05. All analyses were performed in R version 3.5.0.

### Role of the funding source

The funders of the study had no role in study design, data collection, data analysis, data interpretation, or writing of the report. The corresponding author had full access to all the data in the study and had final responsibility for the decision to submit for publication.

## Results

The wave 1, baseline sample comprised 20 743 participants (10 480 female, 10 263 male), who were assessed between April, 1994, and December, 1995. Wave 4 data were collected between January, 2008, and February, 2009, at which point 15 701 (75·7%) participants (8352 women, 7349 men) remained in the sample. Participants who were included in the analysed sample at the wave 4 follow-up were more likely to be female, white, of higher socioeconomic status, and living in urban areas.^30^

We had complete data on wave 1 and wave 4 measures for 6721 (80%) women and 5885 (80%) men, which corresponded to 64% of girls and 57% of boys for whom data were recorded at baseline. Self-reported substance use behaviour was more likely in men than in women at wave 4, as was consuming fast food and soda (table 1). A greater proportion of women reported sexual violence, and women were more likely to have a physical limitation, a diagnosis of depression, and they were less likely to have engaged in physical activity in the past week.

**Table 1:** Descriptive statistics for the Add Health four-wave sample dataset

		**Men (n=5885)**	**Women (n=6721)**
**Wave 1: baseline data**			
Age, years		16·0 (1·8)	15·9 (1·7)
Ethnicity			
	Black	14%	15%
	Asian	4%	4%
	Hispanic	11%	11%
	White	77%	76%
Socioeconomic status		0·11 (1·30)	0·04 (1·32)
Gender expression		0·70 (0·24)	0·70 (0·25)
**Wave 4: health outcomes and behaviours**			
High cholesterol		8%	8%
Obese		36%	37%
High blood pressure		13%	9%
Have used marijuana		62%	53%
Have been diagnosed with depression		10%	23%
Physical limitations		7%	11%
Have been a victim of sexual violence		4%	24%
Have misused prescription drugs		23%	16%
Have used recreational drugs		37%	26%
Have smoked in the past month		42%	34%
Have binge drank in the past year		39%	21%
Consumed soda in the past week		89%	84%
Consumed fast food in the past week		79%	73%
Self-rated health		3·7 (0·9)	3·6 (0·9)
Physical activity		7·1 (6·5)	5·6 (5·2)
Center for Epidemiologic Studies Depression Scale score		8·6 (4·4)	9·3 (4·8)
Heterosexual		93%	80%
Gender expression		0·72 (0·27)	0·75 (0·22)

Data are n (%) or mean (SE) from participants with complete data for wave 1 and wave 4 measures. Means and proportions were adjusted for sample weights, therefore absolute numbers are not reported. Ethnicity categories were not mutually exclusive. We measured socioeconomic status at wave 1 by use of reports of parental education and occupation, household income, and household use of public assistance; this measure was judged on a scale of −5·6 to 3·5. Self-rated health was judged on a scale of 0–5. Physical activity was measured as how many times in the past 7 days that the participant had engaged in activities listed in a series of questions, on a scale of 0–49 (appendix). The Center for Epidemiologic Studies Depression Scale was measured on a scale of 0–60. Gender expression was measured on a scale of 0–1 and was adapted from variables identified by Fleming and colleagues.^22^

We found that for both men and women, adolescent gender expression (wave 1) was significantly predictive of adult gender expression (wave 4; appendix). For each increase of 1 SD in gender expression at wave 1, gender expression at wave 4 increased by 0·14 SD (95% CI 0·12–0·16) for both men and women.

Data on the associations between gender expression and health outcomes and behaviours are shown separately for wave 1 (baseline) gender expression in male participants (table 2) and in female participants (table 3) and for wave 4 gender expression in male participants (table 4) and in female participants (table 5). Model 1 is the linear model; model 2 is the first categorical model, which compares the middle and highest tertiles with the lowest tertile; and model 3 is the second categorical model, which compares the lowest and highest tertiles with the middle tertile. We also created a series of locally weighted smoothing plots (appendix) to show the direction of association for all significant outcomes and approximate effect sizes.

**Table 2:** Association between wave 1 (baseline) gender expression and several wave 4 health outcomes, male participants only

	**Model 1: Linear model**			**Model 2: Middle to lowest**			**Model 2: Highest to lowest**			**Model 3: Lowest to middle**			**Model 3: Highest to middle**		
	β-co-efficient	SE	p	β-co-efficient	SE	p	β-co-efficient	SE	p	β-co-efficient	SE	p	β-co-efficient	SE	p
Obese	0·01	0·04	0·89	−0·01	0·09	0·97	0·03	0·10	0·79	0·01	0·09	0·97	0·03	0·10	0·77
Binary CESD score	0·02	0·05	0·71	−0·10	0·12	0·45	0·02	0·11	0·87	−0·09	0·13	0·45	0·11	0·13	0·38
Physical activity	−0·07	0·11	0·48	−0·62	0·25	0·02	−0·30	0·25	0·23	0·62	0·25	0·02	0·32	0·25	0·21
Self-rated health	0·04	0·02	0·01	0·03	0·04	0·40	0·10	0·04	0·01	0·03	0·04	0·40	0·07	0·04	0·13
Continuous CESD score	0·05	0·07	0·53	−0·25	0·19	0·20	0·17	0·19	0·35	0·25	0·19	0·20	0·43	0·20	0·03
Diagnosed with depression	−0·09	0·06	0·11	−0·23	0·13	0·09	−0·18	0·14	0·17	0·23	0·13	0·09	0·04	0·13	0·73
High cholesterol	−0·11	0·07	0·09	−0·21	0·17	0·21	−0·25	0·16	0·12	0·21	0·17	0·21	−0·04	0·17	0·71
High blood pressure	−0·07	0·05	0·21	0·09	0·13	0·47	−0·25	0·14	0·07	−0·09	0·13	0·47	−0·35	0·12	0·003
Migraine	−0·10	0·06	0·11	−0·21	0·15	0·17	−0·25	0·16	0·12	0·21	0·15	0·17	−0·04	0·17	0·79
Physical limitations	−0·04	0·08	0·65	0·04	0·19	0·83	−0·07	0·17	0·67	−0·04	0·19	0·83	−0·12	0·17	0·49
Have been a victim of sexual violence	0·06	0·10	0·59	−0·08	0·24	0·72	0·28	0·21	0·19	0·08	0·24	0·72	0·36	0·23	0·11
Have misused prescription drugs	0·05	0·05	0·38	0·11	0·11	0·34	0·09	0·12	0·45	−0·11	0·11	0·34	−0·02	0·12	0·86
Have smoked in the past month	0·14	0·04	0·0002	0·16	0·08	0·06	0·33	0·08	0·0002	−0·16	0·08	0·06	0·17	0·09	0·04
Have binge drank in the past year	0·04	0·04	0·34	−0·01	0·10	0·94	0·16	0·11	0·13	0·01	0·10	0·94	0·17	0·10	0·08
Have used marijuana	0·10	0·04	0·01	0·24	0·10	0·02	0·26	0·09	0·01	−0·24	0·10	0·02	0·02	0·10	0·82
Have used recreational drugs	0·10	0·04	0·01	0·16	0·10	0·08	0·27	0·10	0·01	−0·16	0·10	0·08	0·12	0·11	0·29
Consumed soda in the past week	0·11	0·05	0·04	0·01	0·12	0·91	0·12	0·13	0·36	−0·01	0·12	0·91	0·11	0·14	0·43
Consumed fast food in the past week	0·13	0·04	0·0009	0·31	0·11	0·003	0·33	0·11	0·005	−0·31	0·11	0·003	0·02	0·14	0·87

Model 1 uses a linear version of gender expression. Model 2 uses a categorical version of gender expression broken into tertiles, which are then compared with the lowest category, as the reference. Model 3 uses the same categorical version of gender expression as model 2, but with the middle category as the reference. All models include sociodemographic controls, wave 4 gender expression, and wave 1 school-level aggregate gender expression. Migraines were assessed as a binary measure. Physical activity was reported on a scale of 0–49, to indicate the number of times in the past week an individual engaged in several types of physical activity, as asked in a series of questions (appendix). Self-rated health was assessed on a scale of 0–5. The CESD scale was measured on a scale of 0–60. CESD=Center for Epidemiologic Studies Depression Scale.

**Table 3:** Association between wave 1 (baseline) gender expression and several wave 4 health outcomes, female participants only

	**Model 1: Linear model**			**Model 2: Middle to lowest**			**Model 2: Highest to lowest**			**Model 3: Lowest to middle**			**Model 3: Highest to middle**		
	β-co-efficient	SE	p	β-co-efficient	SE	p	β-co-efficient	SE	p	β-co-efficient	SE	p	β-co-efficient	SE	p
Obese	0·04	0·04	0·28	−0·05	0·09	0·55	0·15	0·09	0·09	0·05	0·09	0·55	0·21	0·08	0·01
Binary CESD score	0·04	0·04	0·32	0·01	0·08	0·98	0·06	0·09	0·52	−0·01	0·08	0·98	0·06	0·09	0·52
Physical activity	−0·09	0·09	0·34	−0·02	0·21	0·94	−0·28	0·20	0·18	0·02	0·21	0·94	−0·24	0·17	0·16
Self-rated health	−0·06	0·02	0·0006	−0·05	0·04	0·19	−0·14	0·04	0·0003	0·05	0·04	0·19	−0·09	0·04	0·03
Continuous CESD score	0·10	0·08	0·11	0·06	0·18	0·75	0·19	0·18	0·28	−0·06	0·18	0·75	0·13	0·18	0·46
Diagnosed with depression	0·03	0·05	0·54	−0·10	0·11	0·38	0·14	0·10	0·12	0·10	0·11	0·38	0·24	0·11	0·03
High cholesterol	0·21	0·07	0·0006	0·25	0·16	0·12	0·42	0·15	0·002	−0·25	0·16	0·12	0·17	0·16	0·29
High blood pressure	0·04	0·08	0·62	0·11	0·19	0·55	0·15	0·16	0·35	−0·11	0·19	0·55	0·04	0·13	0·77
Migraine	0·02	0·05	0·96	0·05	0·10	0·6	0·03	0·12	0·77	−0·05	0·10	0·40	−0·02	0·1	0·86
Physical limitations	0·11	0·07	0·09	0·05	0·14	0·74	0·35	0·15	0·02	−0·05	0·14	0·74	0·28	0·13	0·02
Have been a victim of sexual violence	0·19	0·05	0·0001	0·09	0·12	0·43	0·49	0·11	<0·0001	−0·09	0·12	0·43	0·41	0·10	0·0001
Have misused prescription drugs	0·12	0·05	0·02	0·08	0·12	0·49	0·29	0·11	0·01	−0·08	0·12	0·49	0·21	0·10	0·03
Have smoked in the past month	0·03	0·04	0·37	−0·05	0·09	0·57	0·07	0·09	0·42	0·05	0·09	0·57	0·12	0·08	0·10
Have binge drank in the past year	−0·01	0·05	0·92	0·08	0·09	0·42	0·02	0·10	0·86	−0·08	0·09	0·42	−0·06	0·09	0·53
Have used marijuana	0·07	0·03	0·07	−0·01	0·08	0·96	0·17	0·08	0·06	0·008	0·08	0·96	0·17	0·08	0·03
Have used recreational drugs	0·10	0·04	0·005	0·01	0·09	0·96	0·21	0·08	0·02	−0·01	0·09	0·96	0·18	0·08	0·02
Consumed soda in the past week	−0·01	0·05	0·82	−0·11	0·10	0·28	0·10	0·12	0·41	0·11	0·10	0·28	0·21	0·11	0·05
Consumed fast food in the past week	0·01	0·05	0·87	−0·01	0·10	0·93	0·03	0·10	0·71	0·01	0·10	0·93	0·03	0·09	0·66

Model 1 uses a linear version of gender expression. Model 2 uses a categorical version of gender expression broken into tertiles, which are then compared with the lowest category, as the reference. Model 3 uses the same categorical version of gender expression as model 2, but with the middle category as the reference. All models include sociodemographic controls, wave 4 gender expression, and wave 1 school-level aggregate gender expression. Migraines were assessed as a binary measure. Physical activity was reported on a scale of 0–49, to indicate the number of times in the past week an individual engaged in several types of physical activity, as asked in a series of questions (appendix). Self-rated health was assessed on a scale of 0–5. The CESD scale was measured on a scale of 0–60. CESD=Center for Epidemiologic Studies Depression Scale.

**Table 4:** Association between wave 4 gender expression and several wave 4 health outcomes, male participants only

	**Model 1: Linear model**			**Model 2: Middle to lowest**			**Model 2: Highest to lowest**			**Model 3: Lowest to middle**			**Model 3: Highest to middle**		
	β-co-efficient	SE	p	β-co-efficient	SE	p	β-co-efficient	SE	p	β-co-efficient	SE	p	β-co-efficient	SE	p
Obese	0·04	0·04	0·35	0·07	0·09	0·46	0·07	0·09	0·48	−0·07	0·09	0·46	0·01	0·09	0·99
Binary CESD score	−0·15	0·05	0·002	0·03	0·10	0·79	−0·35	0·12	0·005	−0·03	0·10	0·79	−0·37	0·12	0·003
Physical activity	0·28	0·12	0·02	−0·05	0·29	0·87	0·85	0·26	0·001	0·05	0·29	0·87	0·89	0·26	0·0007
Self-rated health	0·04	0·02	0·03	−0·01	0·04	0·94	0·10	0·04	0·03	<0·01	0·04	0·94	0·10	0·04	0·01
Continuous CESD score	−0·39	0·08	<0·0001	−0·11	0·20	0·59	−0·84	0·18	<0·0001	0·11	0·20	0·59	−0·73	0·17	0·0001
Diagnosed with depression	−0·16	0·06	0·01	−0·09	0·18	0·63	−0·31	0·12	0·01	0·09	0·18	0·63	−0·23	0·14	0·10
High cholesterol	−0·17	0·07	0·02	−0·26	0·17	0·13	−0·37	0·16	0·02	0·26	0·17	0·13	−0·11	0·15	0·47
High blood pressure	−0·13	0·06	0·03	−0·31	0·13	0·02	−0·23	0·14	0·1	0·31	0·13	0·02	0·08	0·12	0·53
Migraine	−0·12	0·06	0·07	−0·19	0·15	0·22	−0·31	0·15	0·05	0·19	0·15	0·22	−0·12	0·15	0·43
Physical limitations	−0·17	0·08	0·04	−0·13	0·19	0·50	−0·32	0·18	0·09	0·13	0·19	0·50	−0·19	0·18	0·29
Have been a victim of sexual violence	−0·01	0·09	0·88	−0·09	0·23	0·68	0·03	0·21	0·89	0·09	0·23	0·68	0·12	0·23	0·59
Have misused prescription drugs	0·23	0·05	<0·0001	0·29	0·12	0·02	0·55	0·11	<0·0001	−0·28	0·12	0·02	0·26	0·11	0·02
Have smoked in the past month	0·29	0·04	<0·0001	0·34	0·09	0·0007	0·71	0·09	<0·0001	−0·34	0·09	0·0007	0·37	0·09	0·0001
Have binge drank in the past year	0·30	0·04	<0·0001	0·40	0·11	0·0003	0·71	0·10	<0·0001	−0·40	0·11	0·0003	0·31	0·09	0·0007
Have used marijuana	0·33	0·04	<0·0001	0·35	0·09	0·0002	0·87	0·11	<0·0001	−0·35	0·09	0·0002	0·52	0·10	<0·0001
Have used recreational drugs	0·34	0·05	<0·0001	0·36	0·10	0·0004	0·83	0·11	<0·0001	−0·36	0·10	0·0004	0·46	0·09	<0·0001
Consumed soda in the past week	0·04	0·06	0·54	0·06	0·15	0·67	0·08	0·14	0·58	−0·06	0·15	0·67	0·01	0·15	0·94
Consumed fast food in the past week	−0·05	0·05	0·34	0·08	0·10	0·43	−0·17	0·12	0·16	−0·08	0·10	0·43	−0·25	0·11	0·03

Model 1 uses a linear version of gender expression. Model 2 uses a categorical version of gender expression broken into tertiles, which are then compared with the lowest category, as the reference. Model 3 uses the same categorical version of gender expression as model 2, but with the middle category as the reference. All models include sociodemographic controls, wave 4 gender expression, and wave 1 school-level aggregate gender expression. Migraines were assessed as a binary measure. Physical activity was reported on a scale of 0–49, to indicate the number of times in the past week an individual engaged in several types of physical activity, as asked in a series of questions (appendix). Self-rated health was assessed on a scale of 0–5. The CESD scale was measured on a scale of 0–60. CESD=Center for Epidemiologic Studies Depression Scale.

**Table 5:** Association between wave 4 gender expression and several wave 4 health outcomes, female participants only

	**Model 1: Linear model**			**Model 2: Middle to lowest**			**Model 2: Highest to lowest**			**Model 3: Lowest to middle**			**Model 3: Highest to middle**		
	β-co-efficient	SE	p	β-co-efficient	SE	p	β-co-efficient	SE	p	β-co-efficient	SE	p	β-co-efficient	SE	p
Obese	0·03	0·04	0·36	0·12	0·09	0·16	0·16	0·09	0·09	−0·12	0·09	0·16	0·03	0·09	0·75
Binary CESD score	0·19	0·04	<0·0001	0·10	0·09	0·27	0·60	0·10	<0·0001	−0·10	0·09	0·27	0·51	0·08	<0·0001
Physical activity	−0·35	0·08	<0·0001	−0·36	0·19	0·05	−0·68	0·18	0·0001	0·36	0·19	0·05	−0·36	0·20	0·07
Self-rated health	−0·03	0·01	0·05	−0·03	0·04	0·45	−0·05	0·04	0·22	0·03	0·04	0·45	−0·02	0·04	0·62
Continuous CESD score	0·49	0·09	<0·0001	0·19	0·19	0·31	1·46	0·24	<0·0001	−0·19	0·19	0·32	1·26	0·21	<0·0001
Diagnosed with depression	0·14	0·04	0·003	0·11	0·11	0·34	0·37	0·10	0·0003	−0·11	0·11	0·34	0·26	0·10	0·01
High cholesterol	0·03	0·09	0·72	0·03	0·18	0·87	0·22	0·18	0·22	−0·03	0·18	0·87	0·19	0·13	0·13
High blood pressure	0·09	0·06	0·15	0·16	0·18	0·37	0·33	0·14	0·02	−0·16	0·18	0·37	0·16	0·19	0·37
Migraine	0·05	0·04	0·28	0·11	0·10	0·28	0·21	0·10	0·04	−0·11	0·10	0·28	0·11	0·09	0·23
Physical limitations	0·14	0·07	0·04	−0·03	0·14	0·89	0·43	0·14	0·002	0·03	0·14	0·88	0·47	0·13	0·0005
Have been a victim of sexual violence	−0·14	0·04	0·0003	−0·11	0·09	0·25	−0·19	0·10	0·06	0·11	0·09	0·25	−0·09	0·10	0·39
Have misused prescription drugs	−0·19	0·04	<0·0001	−0·29	0·11	0·01	−0·32	0·11	0·004	0·29	0·11	0·01	−0·03	0·11	0·77
Have smoked in the past month	−0·19	0·03	<0·0001	−0·24	0·08	0·007	−0·38	0·08	<0·0001	0·24	0·08	0·007	−0·14	0·09	0·13
Have binge drank in the past year	−0·21	0·04	<0·0001	−0·09	0·12	0·42	−0·58	0·11	<0·0001	0·09	0·12	0·42	−0·48	0·11	<0·0001
Have used marijuana	−0·18	0·03	<0·0001	−0·25	0·08	0·003	−0·38	0·09	<0·0001	0·25	0·08	0·003	−0·13	0·08	0·12
Have used recreational drugs	−0·25	0·03	<0·0001	−0·27	0·09	0·006	−0·54	0·09	<0·0001	0·27	0·09	0·006	−0·27	0·11	0·01
Consumed soda in the past week	−0·01	0·05	0·84	−0·03	0·11	0·78	−0·06	0·13	0·62	0·03	0·11	0·78	−0·03	0·11	0·77
Consumed fast food in the past week	0·11	0·04	0·01	0·19	0·10	0·05	0·23	0·10	0·02	−0·19	0·10	0·05	0·04	0·11	0·69

Model 1 uses a linear version of gender expression. Model 2 uses a categorical version of gender expression broken into tertiles, which are then compared with the lowest category, as the reference. Model 3 uses the same categorical version of gender expression as model 2, but with the middle category as the reference. All models include sociodemographic controls, wave 4 gender expression, and wave 1 school-level aggregate gender expression. Migraines were assessed as a binary measure. Physical activity was reported on a scale of 0–49, to indicate the number of times in the past week an individual engaged in several types of physical activity, as asked in a series of questions (appendix). Self-rated health was assessed on a scale of 0–5. The CESD scale was measured on a scale of 0–60. CESD=Center for Epidemiologic Studies Depression Scale.

In men, higher wave 1 gender expression (ie, higher masculinity in adolescence) was positively associated with several health behaviours and outcomes in adulthood, including having smoked in the previous 30 days, having ever used marijuana or recreational drugs, fast food and soda consumption in the previous week, better self-rated health, and more physical activity (table 2; appendix). Masculinity in adolescence was negatively associated with adult high blood pressure (model 3, highest to middle tertile comparison only). Most of these relationships were roughly linear; however, men who were at lowest risk of marijuana use were in the lowest (least masculine) gender expression tertile, with a fairly consistent risk across the top two (more masculine) tertiles. Boys in the highest tertile for masculinity during adolescence also reported more depressive symptoms on the CESD scale at adulthood than those in the middle tertile.

In women, higher wave 1 gender expression (ie, higher femininity in adolescence) was positively predictive of many of the health outcomes and behaviours in adulthood that we tested for, including high cholesterol, physical limitations, ever having used recreational drugs, ever having misused prescription drugs, experience of sexual violence, obesity, and depression (model 3, highest to middle comparison only; table 3). Higher femininity in adolescence was negatively associated with self-rated health in adulthood. For some of these outcomes, such as recreational drug use and sexual violence, the risk was primarily found in those at the high (more feminine) end of the gender expression spectrum. The risk of sexual violence was significantly increased for those in the highest tertile versus those in the lowest (least feminine) tertile (model 2), and for those in the highest (most feminine) tertile versus those in the middle tertile (model 3; appendix). When we removed the questions in the gender expression scale that were specific to depressive symptoms, we found that adolescent feminine gender expression was negatively correlated with adult substance use (data not shown).

For men, the association between adult (wave 4) gender expression and health outcomes was similar in direction to that of adolescent (wave 1) gender expression and adult health outcome associations, but stronger; a greater number of health outcomes could be predicted, and effect sizes associated with wave 4 gender expression were greater in magnitude than those of wave 1 gender expression (table 4; appendix). Higher adult (wave 4) masculinity in men was linearly associated with an increased likelihood of several health behaviours in adulthood, namely prescription drug misuse, cigarette smoking, heavy drinking, marijuana use, and recreational drug use, and it was associated with a decreased likelihood at wave 4 of high CESD score, a depression diagnosis, high cholesterol, high blood pressure, and physical limitations, with higher risks for those at the less masculine end of the gender expression spectrum. The most masculine men reported the best self-rated health and the most physical activity in adulthood.

For women, higher adult (wave 4) gender expression was associated with an increased likelihood of many negative health outcomes and behaviours, including fast food consumption, high CESD scores, depression diagnosis, and physical limitations (table 5; appendix). For some outcomes, we saw a reversal of associations relative to what was seen with wave 1 gender expression. For example, unlike results with adolescent (wave 1) gender expression, adult (wave 4) feminine gender expression was negatively associated with adult (wave 4) experiences of sexual violence, prescription drug misuse, smoking, heavy drinking, and marijuana and recreational drug use. For many of the substance use outcomes, except for heavy drinking, the risk was highest for the least feminine women. For sexual violence, a similar pattern was seen, but with a slight increase in risk for those who were most feminine.

In an additional post-hoc analysis, we examined the association between wave 1 and wave 4 gender expression and wave 4 sexual orientation (appendix). For women, wave 4 feminine gender expression was positively associated with the respondent reporting heterosexuality whereas, for men, there was a positive association between heterosexuality and wave 1 masculine gender expression in addition to wave 4 masculine gender expression (appendix). Additional regression models with the inclusion of sexual orientation, however, did not significantly change our results (data not shown).

The high blood pressure biomarker was not significantly associated with wave 1 or wave 4 gender expression for men or women; however, the high cholesterol biomarker results were concordant with the self-report measure (data not shown). Additional analyses revealed that protective associations between masculine gender expression and health for men were not due to men engaging in more physical activity (data not shown).

## Discussion

Our results suggest that gender expression tends to be stable from adolescence (ages 11–18 years) into adulthood (ages 24–32 years) and is strongly associated with important adult health outcomes and behaviours. Gender expression in adolescence was significantly predictive of gender expression in adulthood, both for men and for women. This trend was significant, but the effect sizes were modest, suggesting that, although gender expression has stability over time, gender expression in adolescence is not necessarily deterministic of gender expression in adulthood. This finding is consistent with previous research,^24^ which has suggested that scores on gender scales, such as the Bem Sex Role Inventory, can be affected by cultural change.

We note several important results from our longitudinal health analysis. First, we found that a person's gender expression as an adolescent was associated with their health behaviours and outcomes far into the future. Although it is well established that childhood experiences such as exposure to trauma or neglect can affect adult health,^26^, ^27^ to our knowledge, this is the first analysis to demonstrate that a person's gender expression—their masculinity or femininity—can track through time to affect their adult health, including the likelihood of them engaging in risky or healthy behaviours and experiencing a variety of health outcomes. Whether that effect is protective or harmful depends upon the sex and gender of the individual, their degree of gender expression, and the behaviour in question.

We found that a more masculine gender expression is strongly and consistently associated with substance use among men, and that the effect is independently robust for both adolescent and adult gender expression. Specifically, the higher a man's masculinity as an adolescent, the more likely he is to use harmful substances (ie, alcohol and drugs) as an adult, regardless of how masculine he is as an adult; the same patterns are seen for masculinity in adulthood. In our study, we build on previous findings that men are more likely to engage in substance use to show that, beyond simply being biologically male, men are more likely to use substances when they score higher on masculinity, and that early life masculinity affects the likelihood of substance use behaviour into adulthood.

Masculine gender expression in men was not entirely negative, however. Although adolescent masculinity was only protective against high cholesterol and predicted better self-rated health, adult masculinity was protective against high blood pressure, high cholesterol, diagnoses of depression, migraines, physical limitations, and CESD scores, and adult masculinity was also associated with higher self-rated health and more physical activity. Supplementary analyses suggested that these findings were not due to men engaging in more physical activity. These positive findings, however, could have been skewed by response bias. Men who are more masculine in gender expression could be less willing to acknowledge physical limitations and the symptoms measured in the CESD scale, more likely to rate their health highly, and to exaggerate physical activity. They might also be less likely to go to the doctor, which would reduce the likelihood of reporting a condition that has been diagnosed in a clinical setting.

The findings were more complex for women. Feminine gender expression in female adolescents was only associated with adverse health outcomes in adulthood, including several substance use behaviours. By contrast, more feminine adult gender expression was protective against all substance use behaviours that we assessed, and it was also protective against reported sexual violence. It is worth noting that we created three variations of the gender expression variable, depending on the outcomes it was used to predict. When we removed the questions in the gender expression scale that were specific to depressive symptoms, we found that adolescent feminine gender expression was negatively correlated with adult substance use, which was consistent with our adult gender expression findings. It would seem, therefore, that the dimension of adolescent femininity that is specific to negative affect could be key in these strong and conflicting associations that persist well into adulthood, suggesting that further research is warranted.

The variables used to construct the gender expression measure were carefully selected on the basis of how well they discriminated between men and women (ie, the sexes), and these variables were different at each wave. The adult (wave 4) version of this measure had fewer questions that were specific to depression, suggesting that, although we know depression is more common in women, depressive symptoms are more strongly discriminating between genders in adolescence than in adulthood. Although femininity as a trait might be protective against substance use for women as adults, when they have greater autonomy and are less constrained by peer expectations, the traits specific to being feminine that are salient in adolescence and the experiences they are likely to have in such contexts seem to carry risks into adulthood that are important to identify and attenuate. A previous study^31^ showed that pseudomature behaviour among young teenagers was predictive of negative adult outcomes, including substance use. The authors hypothesised that this association might occur because the behavioural adaptations associated with popularity and so-called coolness as a young adolescent develop in problematic ways over time. Our results provide evidence that the behavioural adaptations necessary to conform to normative adolescent femininity might develop in ways that are similarly problematic. This finding might provide part of the explanation for why, in women, feminine adolescent gender expression was positively associated with reported sexual violence, whereas this association was not found with feminine adult gender expression. We found a similar dynamic in men, in that highly masculine orientation in adolescence was associated with a higher CESD score as an adult, whereas adult masculine orientation was associated with a lower adult CESD score. Again, some aspect of masculinity that is specific to adolescence might effect a negative trajectory into adulthood, but adult masculinity might be protective.

When we investigated the association of gender expression and sexual orientation, we revealed two interesting dimensions to these dynamics. First, our measure of gender expression was associated with sexual orientation; in other words, men with higher scores on masculinity or women with higher scores on femininity were more likely to self-report as heterosexual in wave 4. Including sexual orientation in our models, however, did not notably change any of our findings, suggesting that although gender expression and sexual orientation are associated, the relationships are independent: sexual orientation does not act as a confounder of the association between gender expression and wave 4 health outcomes.

Our study has limitations. Our health measures (except for the biomarker data) were based on self-reporting, including the health outcomes reported at wave 4. As noted, gender expression might skew the direction of responses towards what is normatively acceptable. Also, because our analyses examined gender dynamics over a broad range of health outcomes, the causal pathways are beyond the scope of our analysis. Finally, because the National Longitudinal Study of Adolescent to Adult Health did not include actual measures of perceived gender norms, our gender expression measure is a behavioural proxy that might have missed some of the more subtle aspects of gender in these contexts.

Despite these limitations, we have compelling evidence that in representative high schools across the USA, gender expression in adolescence is strongly correlated with health outcomes in adulthood along many dimensions of health, and the health effects vary between men and women. Although understanding of gendered pathways to health is increasing,^2^, ^3^, ^13^ less is known about how gender affects the health of gender non-conforming individuals.^2^ Interventions designed to address adolescent gender inequality in health are mostly designed and implemented in developing countries, or they are designed specifically to address sexual and relationship behaviour among adolescents.^13^ A new research agenda is needed to further explore contextual (eg, geographical, urban or rural) and intersectional (eg, socioeconomic status, racial) variations in these relationships at the individual and school level, as potentially important moderators. Research is also needed to test interventions on these issues, since the effects of gender expression on health are significant across many health outcomes and persist over time. Gender transformative interventions that include educational and awareness-building activities and that strengthen social support systems for adolescents hold significant potential for simultaneously changing gender norms and improving health, and they warrant further exploration.^13^
