# Supplementary material for: Adolescent gender norms and adult health outcomes in the USA: a prospective cohort study
Source: Lancet Child Adolesc Health. 2019 Aug;3(8):529–38. doi: 10.1016/S2352-4642(19)30160-9 (PMC6686658; doi:10.1016/S2352-4642(19)30160-9)
Supplement: Supplementary appendix [file mmc1.pdf]

THE LANCET
Child & Adolescent Health

## Supplementary appendix

This appendix formed part of the original submission and has been peer reviewed. We post it as supplied by the authors.

Supplement to: Shakya HB, Domingue B, Nagata JM, Cislaghi B, Weber A, Darmstadt GL. Adolescent gender norms and adult health outcomes in the USA: a prospective cohort study. *Lancet Child Adolesc Health* 2019; published online May 30. http://dx.doi.org/10.1016/S2352-4642(19)30160-9.

Supplemental Table 1. Measures and their corresponding questions used to create the GE variables in A) Waves 1, and B) Wave 4

A. Variables used to construct GE measure Wave 1:

| | |
|---|---|
| Frequency of crying | (0 = Never; 4 = Every day) [2] |
| Frequency of playing an active sport | (0 = Not at all; 3 = 5 or more times) [1] |
| Got into a physical fight | (0 = Never; 2 = More than once) |
| How do you think of yourself in terms of weight? | (1 = Very underweight; 5 = Very overweight) |
| How much do you feel that your friends care about you? | (1 = Not at all; 5 = Very much) |
| Hours per week playing video/computer games | (0–99 h) |
| What do you think your chances are of getting an STD? | (1 = Very high; 5 = No chance) |
| How many hours do you spend working for pay | (0–140 h) |
| Have you ever received an out-of-school suspension from school? | (0 = No; 1 = Yes) |
| Frequency of poor appetite | (0 = Never/Rarely; 3 = Most of the time) |
| Frequency of wearing a helmet while cycling | (0 = Never; 4 = Always) |
| Hours per week listening to the radio | (0–99 h) |
| Frequency of doing work around the house | (0 = Not at all; 3 = 5 or more times) |
| Upset by difficult problems | (1 = Strongly agree; 5 = Strongly disagree) |
| How much do you feel adults care about you? | (1 = Not at all; 5 = Very much) |
| Frequency of moodiness | (0 = Never; 4 = Every day) |
| You have a lot to be proud of | (1 = Strongly agree; 5 = Strongly disagree) |
| Have you taken a pledge to remain a virgin until marriage? | (0 = No; 1 = Yes) |
| Frequency of exercise | (0 = Not at all; 3 = 5 or more times) [1] |
| Rely on gut feelings to make decisions | (1 = Strongly agree; 5 = Strongly disagree) |
| Trying to gain/lose/maintain weight? | (1 = Lose; 4 = Nothing) |
| You never get sad | (1 = Strongly agree; 5 = Strongly disagree) [2] |
| How likely is it that you will go to college | (1 = Low; 5 = High) |
| You felt you were just as good as other people | (0 = Never/Rarely; 3 = Most of the time) [2] |
| Frequency wearing a seatbelt in the car | (0 = Never; 4 = Always) |

[1] Omitted for the version used as a predictor for the physical activity scale.

[2] Omitted for the version used as a predictor for the CESD scale, and the depression diagnosis outcome.

B. Variables used to construct GE measure Wave 4:

| | |
|---|---|
| Compared to other people your age, how intelligent are you? | (1 = Moderately below; 6 = Extremely above) |
| I am not interested in other people's problems | (1 = Strongly agree; 5 = Strongly disagree) |
| I like to take risks | (1 = Strongly agree; 5 = Strongly disagree) |
| In the past 24 h, have you participated in vigorous activity long enough to work up a sweat, get your heart thumping, or get out of breath? | (0 = No; 1 = Yes)[1] |
| I get stressed out easily | (1 = Strongly agree; 5 = Strongly disagree) |
| I am not really interested in others. | (1 = Strongly agree; 5 = Strongly disagree) |
| Have you ever used chewing tobacco at least 20 times in your entire life? | (0 = No; 1 = Yes) |
| Have you ever been arrested? | (0 = No; 1 = Yes) |
| When you go outside on a sunny day for more than one hour, how likely are you to use sunscreen or sunblock? | (1 = Very likely; 3 = Unlikely) |
| I have a vivid imagination | (1 = Strongly agree; 5 = Strongly disagree) |
| I don't talk a lot | (1 = Strongly agree; 5 = Strongly disagree) |
| I sympathize with others' feelings | (1 = Strongly agree; 5 = Strongly disagree) |
| In the past seven days, how many times did you participate in gymnastics, weight lifting, or strength training? | (0 = Not at all; 7 = 7 or more times) [1] |
| Hours per week playing video/computer games | (0–105 h) |
| In past 7 days, how many times did you walk for exercise | (0 = Not at all; 7 = 7 or more times) [1] |
| I have frequent mood swings | (1 = Strongly agree; 5 = Strongly disagree) |
| How often do you pray privately? | (0 = Never; 7 = More than once a day) |
| During typical summer week, how many hours do you spend in the sun during the day? | (0–99 h) |
| Have you ever been in the military? | (0 = No; 1 = Yes) |
| In the past 7 days, how many times did you participate in strenuous team sports such as football, soccer, basketball, lacrosse, rugby, field hockey, or ice hockey? | (0 = Not at all; 7 = 7 or more times) [1] |
| In the past 7 days, you felt too tired to do things. | (0 = Never or rarely; 3 = Most or all of the time)[2] |
| I worry about things | (1 = Strongly agree; 5 = Strongly disagree |

[1] Omitted for the version used as a predictor for the physical activity scale.

[2] Omitted for the version used as a predictor for the CESD scale, and the depression diagnosis outcome.

Supplemental Table 2: Wave 4 health and health behavioural outcomes

| | |
|---|---|
| Self-rated health | "In general, how is your health?" Response options were 1: excellent 2: very good 3: good 4: fair 5: poor. We reverse coded this measure so that answer ranged from 1-5, with 5 representing the highest level of health. |
| Physical limitations | The following questions are about activities you might do during a typical day. How much does your health now limit you in these activities: moderate activities, such as moving a table, pushing a vacuum cleaner, bowling, playing golf? 1: Not limited 2: Limited a little 3: Limited a lot. Individuals who responded they were not limited were coded as 0, those who responded limited a little or limited a lot were coded as 1. |
| High cholesterol, self-report | Has a doctor, nurse or other health care provider ever told you that you have or had: high blood cholesterol or triglycerides or lipids? Yes coded as 1, no as 0. |
| High cholesterol, biomarker, measured | Objective high cholesterol was defined as total cholesterol decile corresponding to the proportion of young adults with total cholesterol ≥240 mg/dL in the National Health and Nutrition Examination Surveys. High cholesterol coded as 1, not high cholesterol as 0. |
| High blood pressure, self-report | Has a doctor, nurse or other health care provider ever told you that you have or had: high blood pressure or hypertension {if female add, when you were not pregnant}? Yes coded as 1, no as 0. |
| High blood pressure, measured or taking medications, biomarkerm | High blood pressure was defined as a measured systolic blood pressure ≥140 mmHg or a measured diastolic blood pressure ≥90 mmHg using the average of two measurements 30 seconds apart from a factory calibrated, Microlife BP3MC1-PC-IB oscillometric blood pressure monitor (MicroLife USA, Inc.; Dunedin, FL) or report of current use of medications for hypertension. High blood pressure coded as 1, not high blood pressure as 0. |
| Body mass index, biomarker | Body mass index (BMI) was calculated using the standard formula weight (kilograms) divided by height (meters) squared (BMI = weight/height$^2$). Weight (Health-o-meter 844KL High Capacity Digital Bathroom Scale; Jarden Corporation; Rye, NY) and height (Carpenter's square, steel tape measure) were measured by the interviewer. |
| Depression Diagnosis | Has a doctor, nurse or other health care provider ever told you that you have or had: depression? Yes coded as 1 and no as 0. |
| Center for Epidemiologic Studies ***Depression*** Scale (CESD), continuous 0-30 | Now, think about the past seven days. How often was each of the following things true during the past seven days:<br>You were bothered by things that usually don't bother you.<br>You could not shake off the blues, even with help from your family and your friends.<br>You felt you were just as good as other people. (reverse coded)<br>You had trouble keeping your mind on what you were doing.<br>You felt depressed.<br>You felt that you were too tired to do things.<br>You felt happy. (reverse coded)<br>You enjoyed life. (reverse coded)<br>You felt sad.<br>You felt that people disliked you, during the past seven days.<br>Response options were 0: Never or rarely, 1: Sometimes 2: A lot of the time 3: most of the time or all of the time. |

| | |
|---|---|
| Prescription drug misuse | Have you ever taken any prescription drugs that were not prescribed for you, taken prescription drugs in larger amounts than prescribed, more often than prescribed, for longer periods than prescribed, or taken prescription drugs that you took only for the feeling or experience they caused? Yes coded as 1, no as 0. |
| Recreational drug use | Have you ever used: 1.) steroids, anabolic steroids or 'body building' drugs 2.) cocaine 3.) crystal meth 4.) other types of illegal drugs, such as LSD, PCP, ecstasy, heroin, or mushrooms; or inhalants 5.) Have you ever injected (shot up with a needle) any illegal drug, such as heroin or cocaine? Participants who answered yes to any of these questions were coded as 1, all others 0. |
| Marijuana use | Have you ever used the following drugs: marijuana. Yes coded as 1, no as 0. |
| Smoking | During the past 30 days, on how many days did you smoke cigarettes? One or more days categorized as 1, no days as 0. |
| Drinking | During the past 12 months, on how many days have you been drunk or very high on alcohol? 0: none 1: 1 or 2 days in the past 12 months 2: once a month or less (3 to 12 days in the past 12 months) 3: 2 or 3 days a month 4: 1 or 2 days a week 5: 3 to 5 days a week 6: every day or almost every day. Binary 0 no days reported, otherwise 1. |
| Fast food consumption | How many times in the past seven days did you eat food from a fast food restaurant, such as McDonald's, Burger King, Wendy's, Arby's, Pizza Hut, Taco Bell, or Kentucky Fried Chicken or a local fast food restaurant? |
| Soda | In the past 7 days, how many regular (non-diet) sweetened drinks did you have? Include regular soda, juice drinks, sweetened tea or coffee, energy drinks, flavored water, or other sweetened drinks. |
| Healthy activities, continuous (0-49) | In the past seven days, how many times did you bicycle, skateboard, dance, hike, hunt, or do yard work?<br>In the past seven days, how many times did you roller blade, roller skate, downhill ski, snow board, play racquet sports, or do aerobics?<br>In the past seven days, how many times did you participate in strenuous team sports such as football, soccer, basketball, lacrosse, rugby, field hockey, or ice hockey?<br>In the past seven days, how many times did you participate in individual sports such as running, wrestling, swimming, cross-country skiing, cycle racing, or martial arts?<br>In the past seven days, how many times did you participate in gymnastics, weight lifting, or strength training?<br>In the past seven days, how many times did you play golf, go fishing or bowling, or play softball or baseball?<br>In the past seven days, how many times did you walk for exercise? |
| Sexual violence | "Have you ever been forced, in a non-physical way, to have any type of sexual activity against your will? For example, through verbal pressure, threats of harm or by being given alcohol or drugs? Do not include any experiences with a parent or adult caregiver." And "Have you ever been physically forced to have any type of sexual activity against your will? Do not include any experiences with a parent or adult caregiver." Participants who answered yes to either question were coded 1, those who answered no to both questions were coded as 0. |

Supplemental Table 3: Socioeconomic status variable construction

| | |
|---|---|
| Socioeconomic status (SES) | We measured social origins of Add Health Study members from information about their families collected from Add Health participants' parents at Wave I using reports of parental education, parental occupation, household income, and household receipt of public assistance. These four measures were correlated (r>0.19). We conducted principal components analysis of the measures to produce a factor score. The first principal component, which we use as our index of socioeconomic status, explained 53% of the variance. |

Supplemental Table 4: Sexual Orientation

| | |
|---|---|
| Sexual Orientation | In Wave 4, participants were asked "Please choose the description that best fits how you think about yourself" with answer choices:<br>1.) 100% heterosexual (straight)<br>2.) mostly heterosexual (straight), but somewhat attracted to people of your own sex<br>3.) bisexual that is, attracted to men and women equally<br>4.) mostly homosexual (gay), but somewhat attracted to people of the opposite sex<br>5.) 100% homosexual (gay)<br>6.) not sexually attracted to either males or females<br>Participants were coded as heterosexual if they answered 1, and bisexual or homosexual if they answers 2-5. |

Supplemental Table 5. Linear regression model showing the association of Wave 1 GE with Wave 4 GE including demographic controls

| | Men | | | Women | | |
|---|---|---|---|---|---|---|
| | Beta | SE | P | Beta | SE | P |
| GE Wave 1 | 0.14 | 0.02 | <0.001 | 0.14 | 0.02 | <0.001 |
| School GE Wave 1 | 0.01 | 0.02 | 0.40 | -0.05 | 0.02 | 0.00 |
| SES | -0.03 | 0.02 | 0.17 | 0.00 | 0.01 | 0.65 |
| Asian | -0.23 | 0.09 | 0.01 | 0.01 | 0.07 | 0.89 |
| Hispanic | -0.18 | 0.05 | 0.00 | -0.03 | 0.04 | 0.53 |
| Black | -0.16 | 0.04 | 0.00 | -0.12 | 0.04 | 0.01 |
| Age | -0.04 | 0.01 | 0.00 | 0.03 | 0.01 | 0.02 |

Supplemental Table 6. Logistic regression models showing the bivariate association of GE with Wave 4 sexual orientation

| | Men Wave 1 | | | Women Wave 1 | | | Men Wave 4 | | | Women Wave 4 | | |
|---|---|---|---|---|---|---|---|---|---|---|---|---|
| | Beta | SE | P | Beta | SE | P | Beta | SE | P | Beta | SE | P |
| GE | 0.37 | 0.06 | 0.00 | 0.01 | 0.04 | 0.79 | 0.41 | 0.07 | 0.00 | 0.23 | 0.03 | 0.00 |

Supplemental Table 7: Association with Wave 1 and Wave 4 continuous versions of GE and multiple Wave 4 health outcomes for male and female participants controlling for socio-demographics and sexual orientation.

| | Men | | | Men | | | Women | | | Women | | |
|---|---|---|---|---|---|---|---|---|---|---|---|---|
| | GE Wave 1 | | | GE Wave 4 | | | GE Wave 1 | | | GE Wave 4 | | |
| | Beta | SE | P | Beta | SE | P | Beta | SE | P | Beta | SE | P |
| Obese | 0.01 | 0.04 | 0.77 | 0.02 | 0.04 | 0.64 | 0.04 | 0.04 | 0.29 | 0.05 | 0.03 | 0.14 |
| CESD binary | 0.03 | 0.05 | 0.45 | -0.14 | 0.05 | 0.01 | 0.05 | 0.04 | 0.24 | 0.23 | 0.05 | 0 |
| Physical activity | 0.01 | 0.04 | 0.77 | 0.02 | 0.04 | 0.64 | -0.1 | 0.09 | 0.3 | -0.33 | 0.08 | 0 |
| Self-rated health | 0.04 | 0.02 | 0.01 | 0.04 | 0.02 | 0.02 | -0.06 | 0.02 | 0 | -0.04 | 0.01 | 0.01 |
| CESD continuous | 0.08 | 0.07 | 0.28 | -0.35 | 0.08 | 0 | 0.12 | 0.08 | 0.13 | 0.58 | 0.09 | 0 |
| Depression diagnosis | -0.06 | 0.06 | 0.35 | -0.12 | 0.06 | 0.03 | 0.04 | 0.05 | 0.41 | 0.19 | 0.05 | 0 |
| High cholesterol | -0.11 | 0.06 | 0.08 | -0.17 | 0.07 | 0.02 | 0.21 | 0.07 | 0 | 0.04 | 0.09 | 0.62 |
| High blood pressure | -0.06 | 0.05 | 0.24 | -0.12 | 0.06 | 0.04 | 0.03 | 0.08 | 0.66 | 0.1 | 0.06 | 0.11 |
| Migraine | -0.09 | 0.07 | 0.16 | -0.11 | 0.06 | 0.1 | 0 | 0.05 | 0.98 | 0.06 | 0.04 | 0.17 |
| Physical limitations | -0.04 | 0.08 | 0.65 | -0.17 | 0.08 | 0.04 | 0.11 | 0.07 | 0.1 | 0.15 | 0.06 | 0.02 |
| Sexual violence | 0.09 | 0.1 | 0.37 | 0.02 | 0.09 | 0.83 | 0.18 | 0.05 | 0 | -0.11 | 0.04 | 0.01 |
| Prescription drug misuse | 0.07 | 0.05 | 0.21 | 0.25 | 0.05 | 0 | 0.11 | 0.05 | 0.03 | -0.14 | 0.05 | 0 |
| Smoking | 0.15 | 0.04 | 0 | 0.3 | 0.04 | 0 | 0.03 | 0.04 | 0.5 | -0.17 | 0.03 | 0 |
| Heavy drinking | 0.05 | 0.04 | 0.25 | 0.31 | 0.04 | 0 | -0.01 | 0.05 | 0.75 | -0.17 | 0.04 | 0 |
| Marijuana use | 0.11 | 0.04 | 0 | 0.33 | 0.04 | 0 | 0.06 | 0.03 | 0.09 | -0.15 | 0.03 | 0 |
| Recreational drug use | 0.12 | 0.04 | 0 | 0.36 | 0.05 | 0 | 0.09 | 0.04 | 0.01 | -0.2 | 0.04 | 0 |
| Soda consumption | 0.11 | 0.05 | 0.04 | 0.04 | 0.06 | 0.52 | -0.01 | 0.05 | 0.84 | -0.01 | 0.05 | 0.79 |
| Fast food consumption | 0.12 | 0.04 | 0 | -0.06 | 0.05 | 0.23 | 0.01 | 0.05 | 0.83 | 0.09 | 0.04 | 0.02 |

Supplemental Figure 1

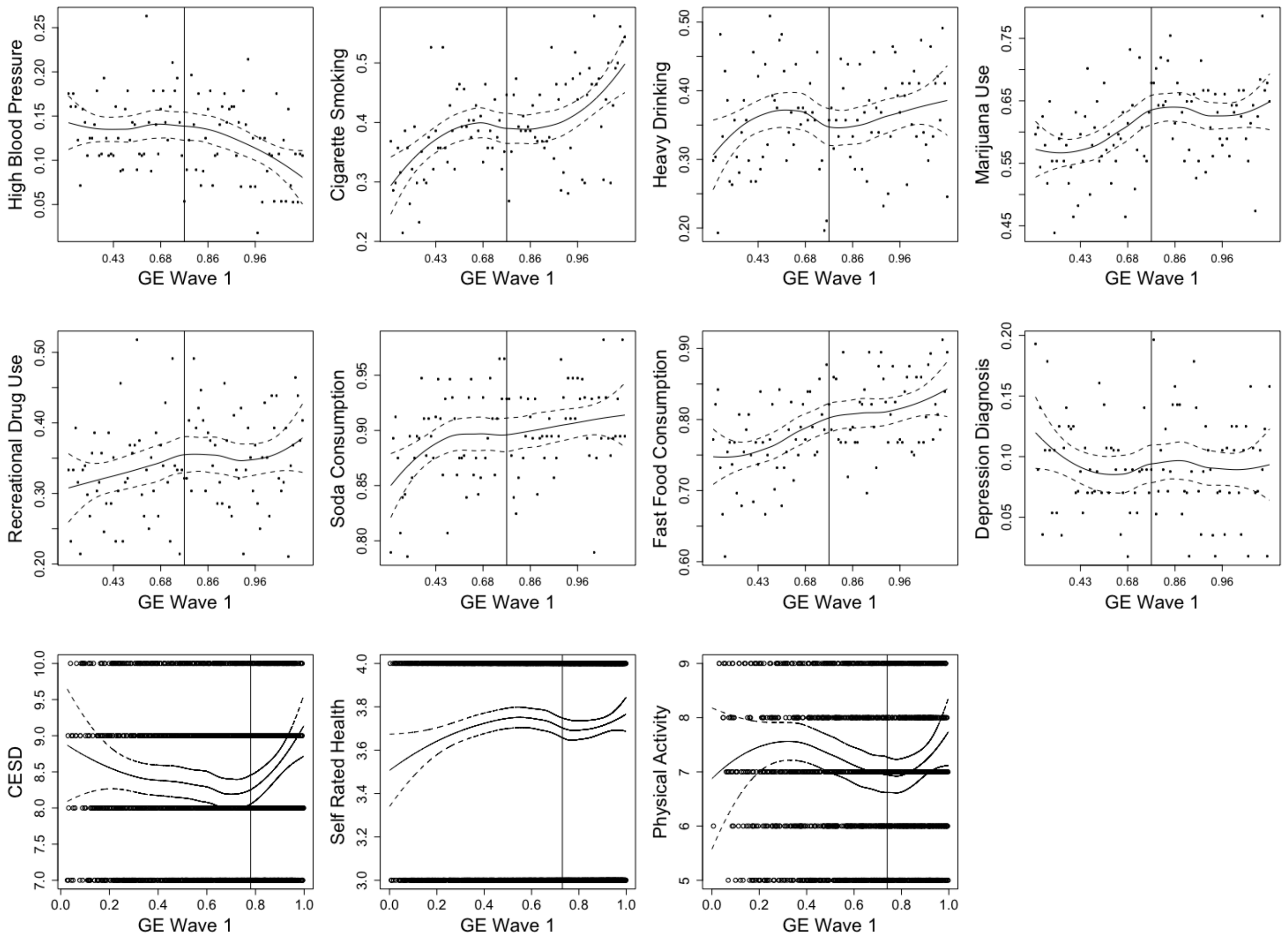


Figure 1a: The association of Wave 1 GE with Wave 4 outcomes among men.

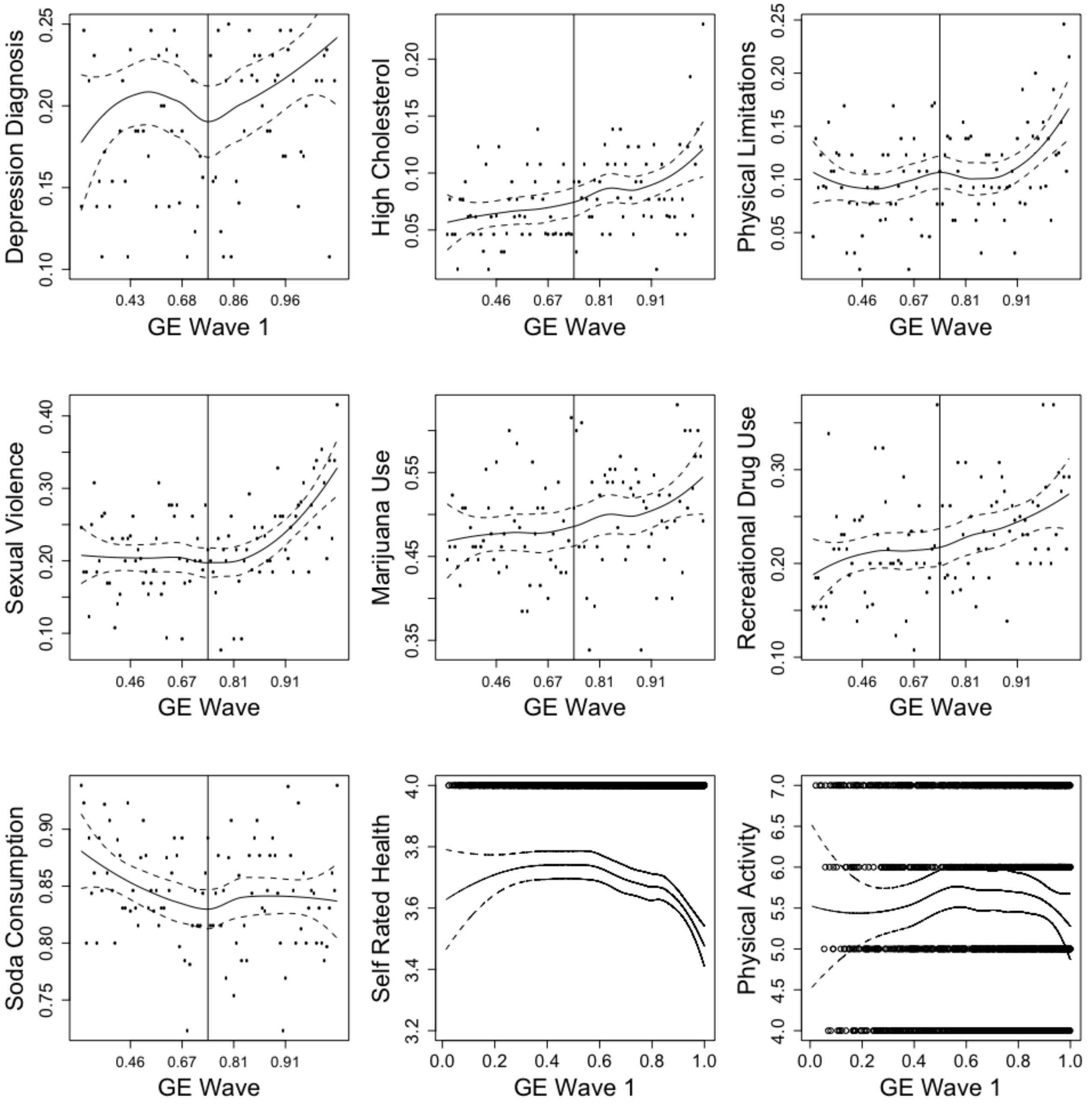


Figure 1b: The association of Wave 1 GE with Wave 4 outcomes among women.

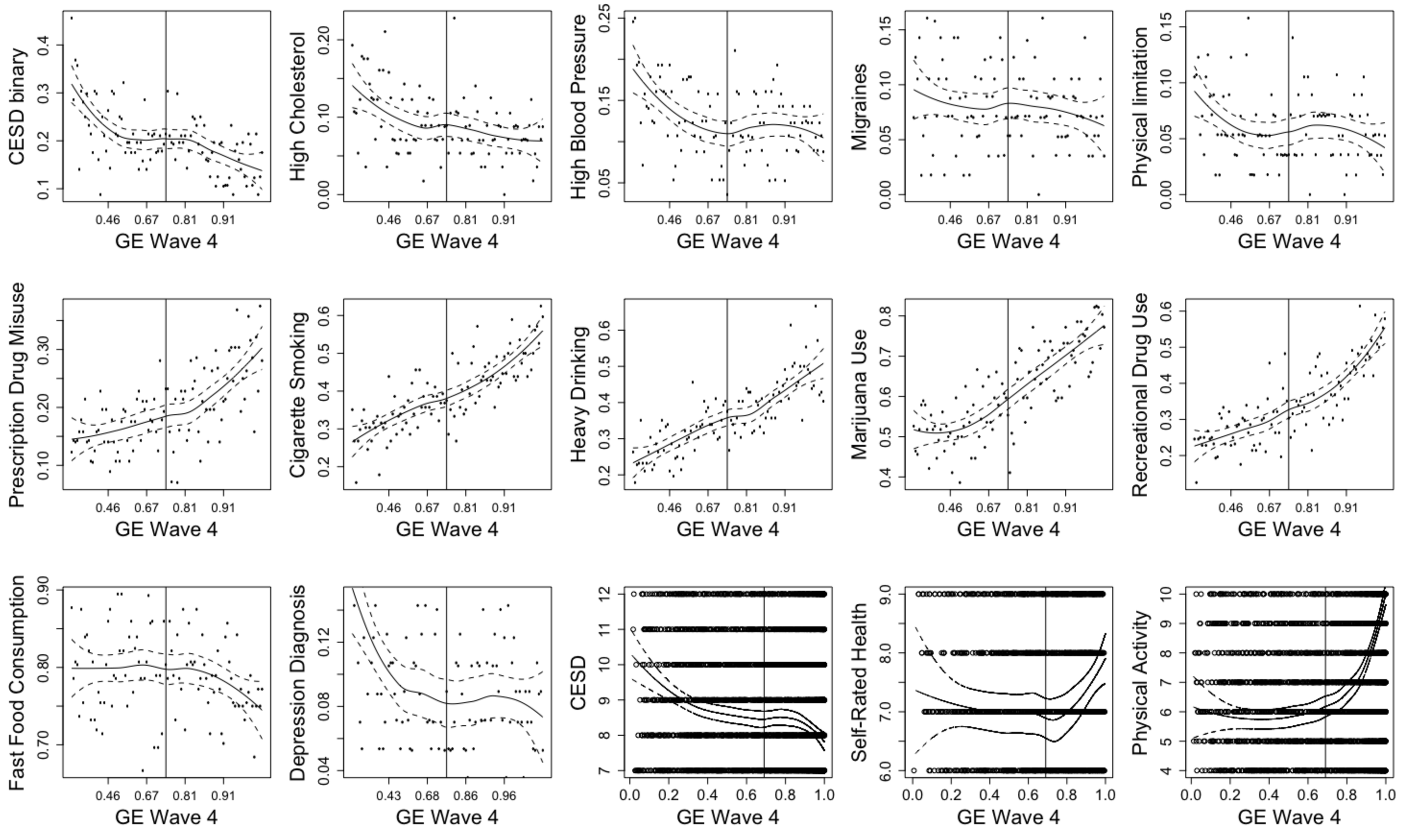


Figure 1c: The association of Wave 4 GE with Wave 4 outcomes among men.

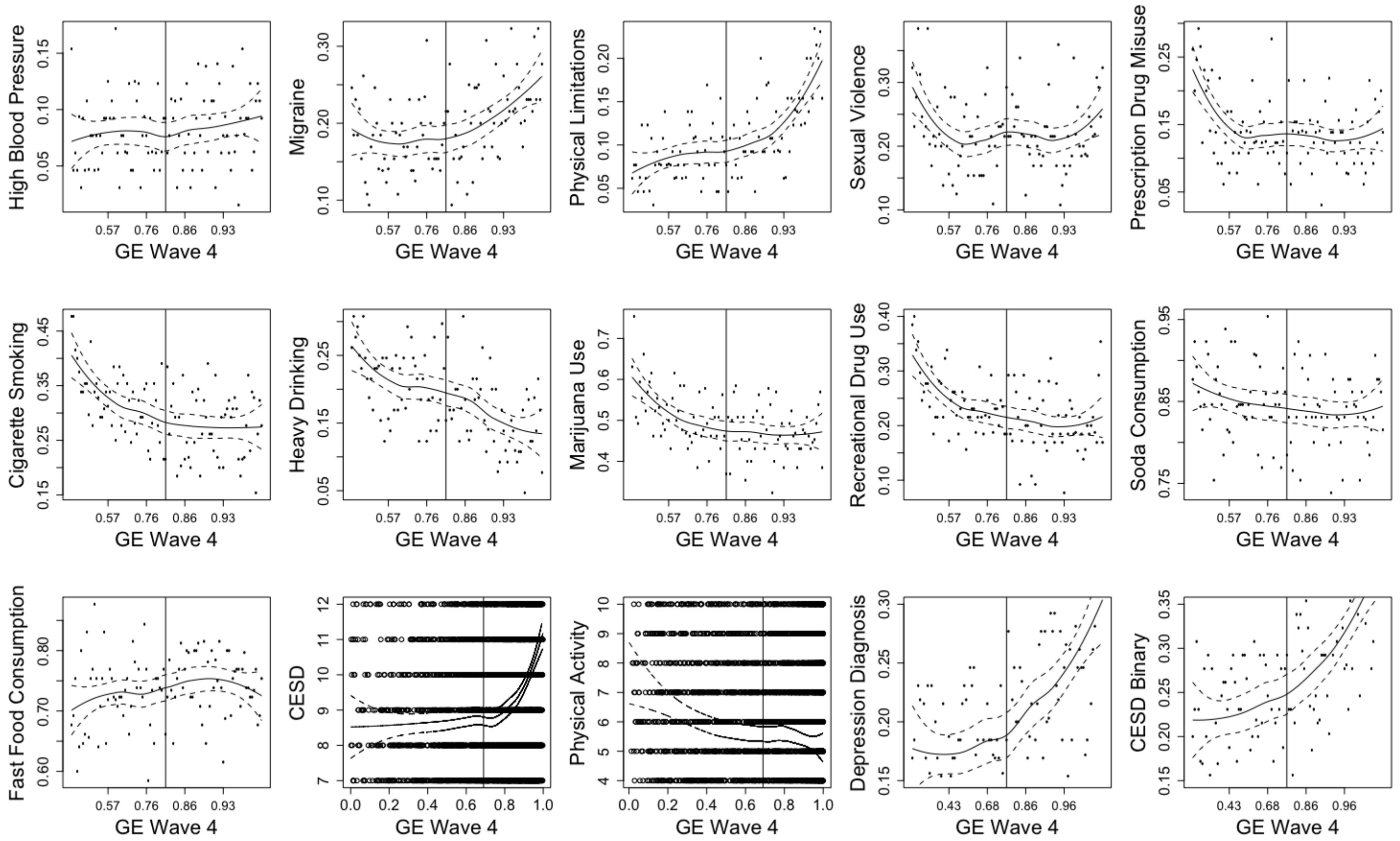


Figure 1d: The association of Wave 4 GE with Wave 4 outcomes among women.
